# Poly[(μ_2_-benzene-1,3-dicarboxyl­ato-κ^2^ *O* ^1^:*O* ^3^){μ_2_-1,2-bis­[(1*H*-imidazol-1-yl)meth­yl]benzene-κ^2^ *N* ^3^:*N* ^3′^}zinc]

**DOI:** 10.1107/S1600536812022544

**Published:** 2012-05-26

**Authors:** Hong Chen, Heng Xu

**Affiliations:** a Department of Chemistry, Chaohu University, Chaohu 238000, People’s Republic of China; b School of Chemistry and Chemical Engineering, Anqing Normal University, Anqing 246003, People’s Republic of China

## Abstract

In the two-dimensional title coordination polymer, [Zn(C_8_H_4_O_4_)(C_14_H_14_N_4_)]_*n*_, the Zn^II^ atom adopts a distorted tetra­hedral geometry, being ligated by two O atoms from two different benzene-1,3-dicarboxyl­ate dianions and two N atoms from two symmetry-related 1,2-bis­(imidazol-1-ylmeth­yl)benzene mol­ecules. The dihedral angles between the imidazole rings and the benzene ring in the neutral ligand are 76.31 (13) and 85.33 (15)°. The Zn^II^ atoms are bridged by dicarboxyl­ate ligands, forming chains parallel to the *a* axis, which are further linked by 1,2-bis­(imidazol-1-ylmeth­yl)benzene mol­ecules, generating a two-dimensional layer structure parallel to the *ac* plane. The crystal structure is enforced by intra­layer and inter­layer C—H⋯O hydrogen bonds.

## Related literature

For background to coordination polymers with bis­(imidazole) ligands, see: Qi *et al.* (2008 ▶); Liu *et al.* (2009 ▶); Hu *et al.* (2008 ▶). For related structures, see: Liu *et al.* (2008 ▶).
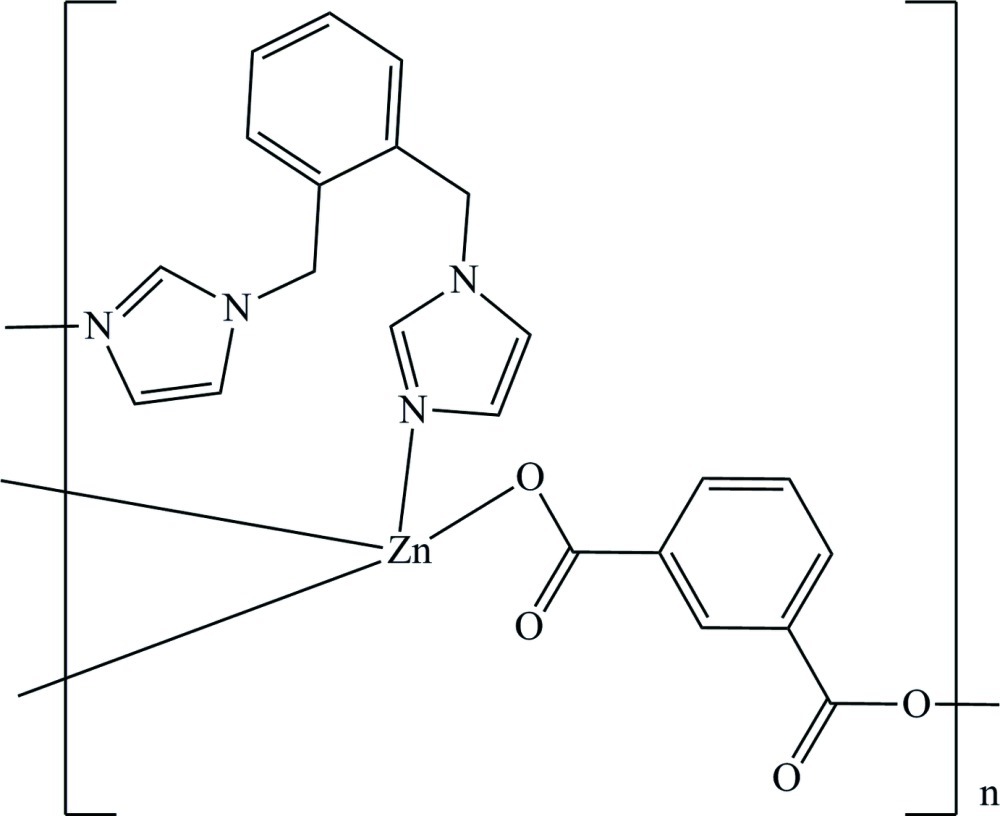


## Experimental

### 

#### Crystal data

[Zn(C_8_H_4_O_4_)(C_14_H_14_N_4_)]
*M* *_r_* = 467.77
Triclinic, 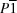

*a* = 10.2028 (14) Å
*b* = 10.2744 (14) Å
*c* = 11.4529 (16) Å
α = 75.405 (2)°
β = 83.480 (2)°
γ = 61.499 (2)°
*V* = 1021.0 (2) Å^3^
*Z* = 2
Mo *K*α radiation
μ = 1.24 mm^−1^
*T* = 293 K
0.26 × 0.24 × 0.20 mm

#### Data collection

Bruker SMART APEX CCD area-detector diffractometer
Absorption correction: multi-scan (*SADABS*; Bruker, 2000 ▶) *T*_min_ = 0.739, *T*_max_ = 0.790
5145 measured reflections
3564 independent reflections
3148 reflections with *I* > 2σ(*I*)
*R*_int_ = 0.062

#### Refinement

*R*[*F* ^2^ > 2σ(*F* ^2^)] = 0.044
*wR*(*F* ^2^) = 0.115
*S* = 1.02
3564 reflections
280 parameters
H-atom parameters constrained
Δρ_max_ = 0.59 e Å^−3^
Δρ_min_ = −0.52 e Å^−3^

### 

Data collection: *SMART* (Bruker, 2000 ▶); cell refinement: *SAINT* (Bruker, 2000 ▶); data reduction: *SAINT*; program(s) used to solve structure: *SHELXTL* (Sheldrick, 2008 ▶); program(s) used to refine structure: *SHELXTL*; molecular graphics: *SHELXTL*; software used to prepare material for publication: *SHELXTL*.

## Supplementary Material

Crystal structure: contains datablock(s) I, global. DOI: 10.1107/S1600536812022544/rz2755sup1.cif

Structure factors: contains datablock(s) I. DOI: 10.1107/S1600536812022544/rz2755Isup2.hkl

Additional supplementary materials:  crystallographic information; 3D view; checkCIF report

## Figures and Tables

**Table 1:** Hydrogen-bond geometry (Å, °)

*D*—H⋯*A*	*D*—H	H⋯*A*	*D*⋯*A*	*D*—H⋯*A*
C9—H9⋯O3^i^	0.93	2.38	3.188 (4)	145
C11—H11⋯O4^ii^	0.93	2.54	3.413 (4)	157
C14—H14⋯O1^ii^	0.93	2.38	3.306 (7)	171
C19—H19*B*⋯O2^iii^	0.97	2.38	3.200 (4)	142
C20—H20⋯O1^iv^	0.93	2.34	3.016 (4)	130
C21—H21⋯O4^v^	0.93	2.54	3.092 (6)	119

Symmetry codes: (i) 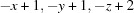; (ii) 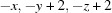; (iii) 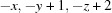; (iv) 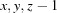; (v) 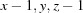.

**Fig. 1.**
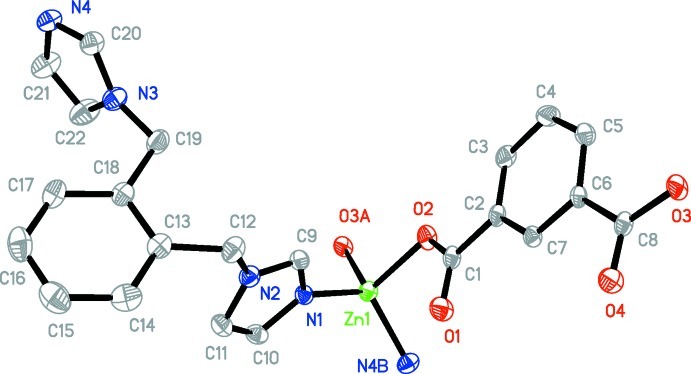
The asymmetric unit of the title compound, extended to show the Zn coordination geometry. Displacement ellipsoids are drawn at the 30% probability level. H atoms have been omitted for clarity. Suffixes A and B denote symmetry operators (-1+x, y, z) and (x, y, 1+z), respectively.

**Fig. 2.**
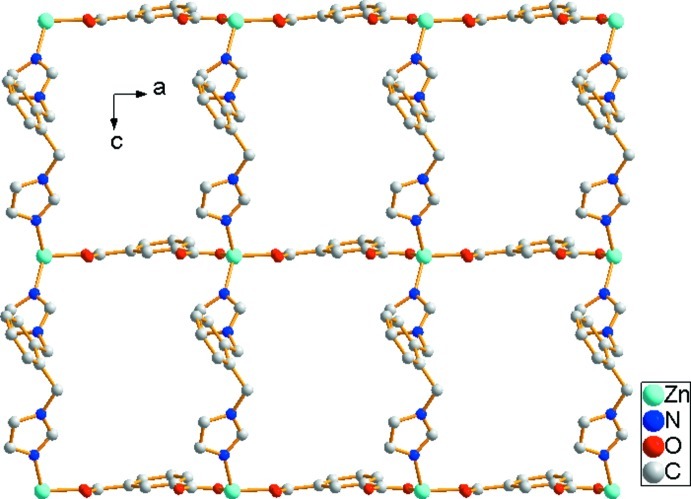
The two-dimensional layer structure of the title compound. Hydrogen atoms are omitted for clarity.

## supplementary crystallographic information

### Comment

Studies of coordination polymers are of considerable interest due to their fascinating network topologies and potential applications in storage, catalysis, molecular magnetism, recognition, and photoluminescence. Recently significant work has been carried out by using metal ions assembly with bis(imidazole) ligands interconnected by flexible spacers (Qi *et al.*, 2008; Liu *et al.*, 2008, 2009). From careful inspection of the reported cases, we found that the ligand exhibits a special ability to formulate the compounds, and different organic anions play an important role in directing the final structures and topologies (Hu *et al.*, 2008). Inspired by the these considerations, 1,2-bis(imidazol-1-ylmethyl)benzene was chosen as neutral ligands, and benzene-1,3-dicarboxylate as co-ligand to construct the title complex.

The title compound is a two-dimensional layer coordination polymer. The zinc(II) atom adopts a distorted tetrahedral geometry, being ligated by two O atoms from two different benzene-1,3-dicarboxylate ligands and two N atoms from two 1,2-bis(imidazol-1-ylmethyl)benzene ligands, as shown in Fig. 1. In the neutral ligand, the N1/N2/C9-C11 and N3/N4/C20/C21 imidazole rings form a dihedral angle of 76.10 (13)° and are tilted by 76.31 (13) and 85.33 (15)° with respect to the benzene ring plane. The Zn atoms are bridged by benzene-1,3-dicarboxylate dianions to form one-dimensional chains running parallel to the *a* axis, which are further linked by 1,2-bis(imidazol-1-ylmethyl)benzene molecules to generate a two-dimensional layer structure parallel to the *ac* plane (Fig. 2). The crystal structure is enforced by intralayer and interlayer C—H···O hydrogen bonds (Table 1).

### Experimental

A mixture of Zn(NO_3_)_2._6H_2_O (29.1 mg, 0.1 mmol), benzene-1,3-dicarboxylate acid (16.4 mg, 0.1 mmol), 1,2-bis(imidazol-1-ylmethyl)benzene (23.8 mg, 0.1 mmol), NaOH (8 mg, 0.2 mmol) and H_2_O (15 ml) was added in a Teflon-lined stainless steel vessel. The vessel was sealed and heated for 3 d at 140 °C. After the mixture was slowly cooled to room temperature, colourless block crystals were obtained in a yield of *ca* 59% based on Zn.

### Refinement

H atoms were positioned geometrically, with C—H = 0.93- 0.97 Å, and constrained to ride on their parent atoms, with *U*_iso_(H) = 1.2 *U*_eq_(C).

### Crystal data

**Table:**

[Zn(C_8_H_4_O_4_)(C_14_H_14_N_4_)]	*Z* = 2
*M**_r_* = 467.77	*F*(000) = 480
Triclinic, *P*1	*D*_x_ = 1.522 Mg m^−^^3^
Hall symbol: -P 1	Mo *K*α radiation, λ = 0.71073 Å
*a* = 10.2028 (14) Å	Cell parameters from 2793 reflections
*b* = 10.2744 (14) Å	θ = 2.3–27.9°
*c* = 11.4529 (16) Å	µ = 1.24 mm^−^^1^
α = 75.405 (2)°	*T* = 293 K
β = 83.480 (2)°	Block, colourless
γ = 61.499 (2)°	0.26 × 0.24 × 0.20 mm
*V* = 1021.0 (2) Å^3^	

### Data collection

**Table:**

Bruker SMART APEX CCD area-detector diffractometer	3564 independent reflections
Radiation source: sealed tube	3148 reflections with *I* > 2σ(*I*)
Graphite monochromator	*R*_int_ = 0.062
phi and ω scans	θ_max_ = 25.1°, θ_min_ = 2.3°
Absorption correction: multi-scan (*SADABS*; Bruker, 2000)	*h* = −12→12
*T*_min_ = 0.739, *T*_max_ = 0.790	*k* = −12→11
5145 measured reflections	*l* = −12→13

### Refinement

**Table:**

Refinement on *F*^2^	Primary atom site location: structure-invariant direct methods
Least-squares matrix: full	Secondary atom site location: difference Fourier map
*R*[*F*^2^ > 2σ(*F*^2^)] = 0.044	Hydrogen site location: inferred from neighbouring sites
*wR*(*F*^2^) = 0.115	H-atom parameters constrained
*S* = 1.02	*w* = 1/[σ^2^(*F*_o_^2^) + (0.0534*P*)^2^ + 0.6759*P*] where *P* = (*F*_o_^2^ + 2*F*_c_^2^)/3
3564 reflections	(Δ/σ)_max_ < 0.001
280 parameters	Δρ_max_ = 0.59 e Å^−^^3^
0 restraints	Δρ_min_ = −0.52 e Å^−^^3^

### Special details

**Table:**

Geometry. All e.s.d.'s (except the e.s.d. in the dihedral angle between two l.s. planes) are estimated using the full covariance matrix. The cell e.s.d.'s are taken into account individually in the estimation of e.s.d.'s in distances, angles and torsion angles; correlations between e.s.d.'s in cell parameters are only used when they are defined by crystal symmetry. An approximate (isotropic) treatment of cell e.s.d.'s is used for estimating e.s.d.'s involving l.s. planes.
Refinement. Refinement of *F*^2^ against ALL reflections. The weighted *R*-factor *wR* and goodness of fit *S* are based on *F*^2^, conventional *R*-factors *R* are based on *F*, with *F* set to zero for negative *F*^2^. The threshold expression of *F*^2^ > σ(*F*^2^) is used only for calculating *R*-factors(gt) *etc*. and is not relevant to the choice of reflections for refinement. *R*-factors based on *F*^2^ are statistically about twice as large as those based on *F*, and *R*- factors based on ALL data will be even larger.

### Fractional atomic coordinates and isotropic or equivalent isotropic displacement parameters (Å^2^)

**Table:**

	*x*	*y*	*z*	*U*_iso_*/*U*_eq_	
Zn1	−0.12836 (4)	0.60720 (4)	1.18865 (3)	0.03331 (15)	
N1	−0.1733 (3)	0.7657 (3)	1.0358 (2)	0.0304 (6)	
N2	−0.1374 (3)	0.8927 (3)	0.8612 (2)	0.0342 (6)	
N3	−0.1584 (3)	0.7322 (3)	0.5120 (3)	0.0420 (7)	
N4	−0.1780 (3)	0.6676 (3)	0.3492 (3)	0.0394 (7)	
O1	0.1045 (3)	0.6829 (3)	1.1935 (2)	0.0464 (6)	
O2	0.0904 (2)	0.4788 (3)	1.1805 (2)	0.0392 (6)	
O3	0.7971 (2)	0.4629 (3)	1.1786 (2)	0.0352 (5)	
O4	0.5906 (3)	0.6760 (3)	1.1811 (2)	0.0400 (6)	
C1	0.1634 (3)	0.5512 (4)	1.1811 (3)	0.0329 (7)	
C2	0.3288 (3)	0.4676 (4)	1.1651 (3)	0.0294 (7)	
C3	0.3983 (4)	0.3254 (4)	1.1412 (3)	0.0366 (8)	
H3	0.3422	0.2771	1.1368	0.044*	
C4	0.5505 (4)	0.2531 (4)	1.1234 (3)	0.0435 (9)	
H4	0.5964	0.1573	1.1060	0.052*	
C5	0.6341 (4)	0.3236 (4)	1.1315 (3)	0.0354 (7)	
H5	0.7365	0.2752	1.1194	0.042*	
C6	0.5668 (3)	0.4654 (3)	1.1575 (3)	0.0273 (6)	
C7	0.4142 (3)	0.5373 (4)	1.1729 (3)	0.0288 (7)	
H7	0.3680	0.6340	1.1887	0.035*	
C8	0.6550 (3)	0.5425 (4)	1.1731 (3)	0.0292 (7)	
C9	−0.0785 (3)	0.7650 (4)	0.9477 (3)	0.0338 (7)	
H9	0.0174	0.6858	0.9458	0.041*	
C10	−0.2999 (4)	0.9013 (4)	1.0029 (3)	0.0362 (7)	
H10	−0.3871	0.9332	1.0477	0.043*	
C11	−0.2793 (4)	0.9816 (4)	0.8960 (3)	0.0400 (8)	
H11	−0.3474	1.0774	0.8544	0.048*	
C12	−0.0539 (4)	0.9346 (4)	0.7587 (3)	0.0403 (8)	
H12A	0.0289	0.8427	0.7412	0.048*	
H12B	−0.0129	0.9931	0.7809	0.048*	
C13	−0.1483 (4)	1.0259 (4)	0.6473 (3)	0.0363 (8)	
C14	−0.2102 (5)	1.1822 (4)	0.6227 (4)	0.0497 (9)	
H14	−0.1920	1.2278	0.6752	0.060*	
C15	−0.2984 (5)	1.2723 (5)	0.5217 (4)	0.0604 (11)	
H15	−0.3391	1.3776	0.5059	0.072*	
C16	−0.3248 (5)	1.2046 (5)	0.4458 (4)	0.0608 (11)	
H16	−0.3853	1.2643	0.3781	0.073*	
C17	−0.2638 (4)	1.0504 (5)	0.4677 (3)	0.0493 (9)	
H17	−0.2820	1.0065	0.4137	0.059*	
C18	−0.1755 (4)	0.9577 (4)	0.5683 (3)	0.0352 (7)	
C19	−0.1066 (4)	0.7864 (4)	0.5940 (3)	0.0443 (9)	
H19A	0.0010	0.7444	0.5880	0.053*	
H19B	−0.1297	0.7492	0.6761	0.053*	
C20	−0.0952 (4)	0.6950 (4)	0.4103 (3)	0.0402 (8)	
H20	−0.0031	0.6888	0.3846	0.048*	
C21	−0.3031 (5)	0.6908 (5)	0.4175 (4)	0.0549 (11)	
H21	−0.3831	0.6807	0.3974	0.066*	
C22	−0.2927 (5)	0.7305 (5)	0.5184 (4)	0.0589 (11)	
H22	−0.3624	0.7523	0.5799	0.071*	

### Atomic displacement parameters (Å^2^)

**Table:**

	*U*^11^	*U*^22^	*U*^33^	*U*^12^	*U*^13^	*U*^23^
Zn1	0.0290 (2)	0.0372 (2)	0.0396 (2)	−0.01929 (18)	−0.00090 (16)	−0.00993 (17)
N1	0.0260 (13)	0.0318 (14)	0.0338 (14)	−0.0133 (11)	−0.0016 (11)	−0.0075 (11)
N2	0.0324 (15)	0.0395 (15)	0.0329 (15)	−0.0183 (12)	0.0007 (11)	−0.0092 (12)
N3	0.0473 (18)	0.0478 (17)	0.0412 (17)	−0.0280 (15)	0.0012 (14)	−0.0152 (14)
N4	0.0390 (16)	0.0522 (18)	0.0384 (16)	−0.0280 (14)	0.0070 (13)	−0.0182 (14)
O1	0.0284 (12)	0.0502 (16)	0.0675 (17)	−0.0200 (12)	−0.0001 (12)	−0.0209 (13)
O2	0.0258 (11)	0.0460 (14)	0.0532 (15)	−0.0237 (11)	−0.0039 (10)	−0.0068 (11)
O3	0.0211 (11)	0.0402 (13)	0.0515 (14)	−0.0187 (10)	0.0002 (10)	−0.0128 (11)
O4	0.0328 (12)	0.0372 (14)	0.0594 (15)	−0.0217 (11)	0.0000 (11)	−0.0149 (11)
C1	0.0252 (16)	0.044 (2)	0.0337 (17)	−0.0203 (15)	−0.0024 (13)	−0.0053 (15)
C2	0.0244 (15)	0.0409 (18)	0.0284 (16)	−0.0203 (14)	−0.0020 (12)	−0.0054 (13)
C3	0.0340 (18)	0.048 (2)	0.0425 (19)	−0.0286 (16)	−0.0001 (14)	−0.0141 (16)
C4	0.0371 (19)	0.045 (2)	0.060 (2)	−0.0215 (16)	0.0027 (17)	−0.0268 (18)
C5	0.0242 (16)	0.0418 (19)	0.0441 (19)	−0.0166 (15)	0.0013 (14)	−0.0140 (15)
C6	0.0223 (15)	0.0335 (16)	0.0291 (16)	−0.0161 (13)	−0.0036 (12)	−0.0038 (13)
C7	0.0239 (15)	0.0309 (16)	0.0347 (17)	−0.0151 (13)	−0.0014 (13)	−0.0067 (13)
C8	0.0258 (16)	0.0408 (19)	0.0280 (16)	−0.0222 (15)	−0.0008 (12)	−0.0050 (13)
C9	0.0266 (16)	0.0360 (18)	0.0377 (18)	−0.0119 (14)	−0.0025 (14)	−0.0111 (15)
C10	0.0299 (17)	0.0364 (18)	0.0429 (19)	−0.0150 (15)	0.0055 (14)	−0.0134 (15)
C11	0.0320 (18)	0.0350 (18)	0.047 (2)	−0.0111 (15)	−0.0015 (15)	−0.0086 (15)
C12	0.0342 (18)	0.060 (2)	0.0368 (18)	−0.0294 (17)	0.0026 (14)	−0.0127 (16)
C13	0.0354 (18)	0.045 (2)	0.0365 (18)	−0.0272 (16)	0.0069 (14)	−0.0084 (15)
C14	0.057 (2)	0.050 (2)	0.055 (2)	−0.036 (2)	0.0094 (19)	−0.0143 (19)
C15	0.065 (3)	0.039 (2)	0.070 (3)	−0.026 (2)	0.004 (2)	0.002 (2)
C16	0.062 (3)	0.059 (3)	0.048 (2)	−0.027 (2)	−0.010 (2)	0.011 (2)
C17	0.058 (2)	0.059 (3)	0.0331 (19)	−0.030 (2)	−0.0040 (17)	−0.0056 (17)
C18	0.0381 (18)	0.0412 (19)	0.0303 (17)	−0.0231 (16)	0.0036 (14)	−0.0063 (14)
C19	0.053 (2)	0.047 (2)	0.0383 (19)	−0.0253 (18)	−0.0073 (16)	−0.0114 (16)
C20	0.0371 (19)	0.048 (2)	0.043 (2)	−0.0243 (17)	0.0016 (16)	−0.0150 (17)
C21	0.054 (2)	0.086 (3)	0.051 (2)	−0.050 (2)	0.0143 (19)	−0.029 (2)
C22	0.059 (3)	0.090 (3)	0.052 (2)	−0.050 (3)	0.019 (2)	−0.030 (2)

### Geometric parameters (Å, º)

**Table:**

Zn1—N1	1.990 (3)	C5—H5	0.9300
Zn1—O2	1.984 (2)	C6—C7	1.380 (4)
Zn1—O3^i^	1.993 (2)	C6—C8	1.502 (4)
Zn1—N4^ii^	2.022 (3)	C7—H7	0.9300
N1—C9	1.315 (4)	C9—H9	0.9300
N1—C10	1.371 (4)	C10—C11	1.350 (5)
N2—C9	1.338 (4)	C10—H10	0.9300
N2—C11	1.370 (4)	C11—H11	0.9300
N2—C12	1.469 (4)	C12—C13	1.501 (5)
N3—C20	1.318 (4)	C12—H12A	0.9700
N3—C22	1.372 (5)	C12—H12B	0.9700
N3—C19	1.459 (4)	C13—C14	1.380 (5)
N4—C20	1.320 (4)	C13—C18	1.396 (5)
N4—C21	1.369 (4)	C14—C15	1.380 (6)
N4—Zn1^iii^	2.022 (3)	C14—H14	0.9300
O1—C1	1.231 (4)	C15—C16	1.360 (6)
O2—C1	1.281 (4)	C15—H15	0.9300
O3—C8	1.280 (4)	C16—C17	1.362 (6)
O3—Zn1^iv^	1.993 (2)	C16—H16	0.9300
O4—C8	1.228 (4)	C17—C18	1.382 (5)
C1—C2	1.498 (4)	C17—H17	0.9300
C2—C3	1.372 (5)	C18—C19	1.511 (5)
C2—C7	1.388 (4)	C19—H19A	0.9700
C3—C4	1.382 (5)	C19—H19B	0.9700
C3—H3	0.9300	C20—H20	0.9300
C4—C5	1.380 (5)	C21—C22	1.349 (6)
C4—H4	0.9300	C21—H21	0.9300
C5—C6	1.379 (4)	C22—H22	0.9300
			
N1—Zn1—O2	102.90 (10)	N2—C9—H9	124.3
N1—Zn1—O3^i^	110.16 (10)	C11—C10—N1	110.0 (3)
O2—Zn1—O3^i^	101.36 (9)	C11—C10—H10	125.0
N1—Zn1—N4^ii^	120.15 (11)	N1—C10—H10	125.0
O2—Zn1—N4^ii^	108.84 (11)	C10—C11—N2	105.8 (3)
O3^i^—Zn1—N4^ii^	111.41 (10)	C10—C11—H11	127.1
C9—N1—C10	105.4 (3)	N2—C11—H11	127.1
C9—N1—Zn1	125.4 (2)	N2—C12—C13	112.8 (3)
C10—N1—Zn1	129.0 (2)	N2—C12—H12A	109.0
C9—N2—C11	107.3 (3)	C13—C12—H12A	109.0
C9—N2—C12	124.5 (3)	N2—C12—H12B	109.0
C11—N2—C12	127.7 (3)	C13—C12—H12B	109.0
C20—N3—C22	107.2 (3)	H12A—C12—H12B	107.8
C20—N3—C19	126.6 (3)	C14—C13—C18	119.2 (3)
C22—N3—C19	125.7 (3)	C14—C13—C12	118.6 (3)
C20—N4—C21	105.0 (3)	C18—C13—C12	122.1 (3)
C20—N4—Zn1^iii^	126.6 (2)	C13—C14—C15	121.3 (4)
C21—N4—Zn1^iii^	128.4 (2)	C13—C14—H14	119.4
C1—O2—Zn1	112.6 (2)	C15—C14—H14	119.4
C8—O3—Zn1^iv^	104.77 (19)	C16—C15—C14	118.9 (4)
O1—C1—O2	123.4 (3)	C16—C15—H15	120.5
O1—C1—C2	119.9 (3)	C14—C15—H15	120.5
O2—C1—C2	116.6 (3)	C15—C16—C17	120.8 (4)
C3—C2—C7	118.9 (3)	C15—C16—H16	119.6
C3—C2—C1	122.2 (3)	C17—C16—H16	119.6
C7—C2—C1	118.9 (3)	C16—C17—C18	121.4 (4)
C2—C3—C4	120.8 (3)	C16—C17—H17	119.3
C2—C3—H3	119.6	C18—C17—H17	119.3
C4—C3—H3	119.6	C17—C18—C13	118.3 (3)
C3—C4—C5	119.7 (3)	C17—C18—C19	122.2 (3)
C3—C4—H4	120.2	C13—C18—C19	119.5 (3)
C5—C4—H4	120.2	N3—C19—C18	113.3 (3)
C6—C5—C4	120.5 (3)	N3—C19—H19A	108.9
C6—C5—H5	119.8	C18—C19—H19A	108.9
C4—C5—H5	119.8	N3—C19—H19B	108.9
C5—C6—C7	119.1 (3)	C18—C19—H19B	108.9
C5—C6—C8	122.0 (3)	H19A—C19—H19B	107.7
C7—C6—C8	118.9 (3)	N3—C20—N4	112.2 (3)
C2—C7—C6	121.0 (3)	N3—C20—H20	123.9
C2—C7—H7	119.5	N4—C20—H20	123.9
C6—C7—H7	119.5	C22—C21—N4	109.7 (3)
O4—C8—O3	122.7 (3)	C22—C21—H21	125.2
O4—C8—C6	120.2 (3)	N4—C21—H21	125.2
O3—C8—C6	117.1 (3)	C21—C22—N3	106.0 (3)
N1—C9—N2	111.5 (3)	C21—C22—H22	127.0
N1—C9—H9	124.3	N3—C22—H22	127.0
			
O2—Zn1—N1—C9	8.3 (3)	C12—N2—C9—N1	172.1 (3)
O3^i^—Zn1—N1—C9	−99.1 (3)	C9—N1—C10—C11	−0.7 (4)
N4^ii^—Zn1—N1—C9	129.4 (2)	Zn1—N1—C10—C11	174.3 (2)
O2—Zn1—N1—C10	−165.8 (3)	N1—C10—C11—N2	0.7 (4)
O3^i^—Zn1—N1—C10	86.8 (3)	C9—N2—C11—C10	−0.5 (4)
N4^ii^—Zn1—N1—C10	−44.7 (3)	C12—N2—C11—C10	−172.2 (3)
N1—Zn1—O2—C1	63.6 (2)	C9—N2—C12—C13	148.5 (3)
O3^i^—Zn1—O2—C1	177.6 (2)	C11—N2—C12—C13	−41.2 (5)
N4^ii^—Zn1—O2—C1	−64.9 (2)	N2—C12—C13—C14	96.4 (4)
Zn1—O2—C1—O1	4.6 (4)	N2—C12—C13—C18	−83.5 (4)
Zn1—O2—C1—C2	−175.2 (2)	C18—C13—C14—C15	0.1 (5)
O1—C1—C2—C3	−174.6 (3)	C12—C13—C14—C15	−179.8 (3)
O2—C1—C2—C3	5.2 (5)	C13—C14—C15—C16	0.3 (6)
O1—C1—C2—C7	4.3 (5)	C14—C15—C16—C17	−0.9 (7)
O2—C1—C2—C7	−175.8 (3)	C15—C16—C17—C18	1.1 (7)
C7—C2—C3—C4	−0.9 (5)	C16—C17—C18—C13	−0.7 (6)
C1—C2—C3—C4	178.1 (3)	C16—C17—C18—C19	−179.9 (4)
C2—C3—C4—C5	1.0 (6)	C14—C13—C18—C17	0.1 (5)
C3—C4—C5—C6	0.2 (5)	C12—C13—C18—C17	−180.0 (3)
C4—C5—C6—C7	−1.3 (5)	C14—C13—C18—C19	179.3 (3)
C4—C5—C6—C8	177.0 (3)	C12—C13—C18—C19	−0.8 (5)
C3—C2—C7—C6	−0.3 (5)	C20—N3—C19—C18	92.4 (4)
C1—C2—C7—C6	−179.3 (3)	C22—N3—C19—C18	−78.1 (5)
C5—C6—C7—C2	1.4 (5)	C17—C18—C19—N3	−7.6 (5)
C8—C6—C7—C2	−176.9 (3)	C13—C18—C19—N3	173.3 (3)
Zn1^iv^—O3—C8—O4	−4.1 (4)	C22—N3—C20—N4	−0.5 (5)
Zn1^iv^—O3—C8—C6	176.6 (2)	C19—N3—C20—N4	−172.4 (3)
C5—C6—C8—O4	170.8 (3)	C21—N4—C20—N3	0.6 (4)
C7—C6—C8—O4	−10.8 (5)	Zn1^iii^—N4—C20—N3	179.9 (2)
C5—C6—C8—O3	−9.9 (5)	C20—N4—C21—C22	−0.4 (5)
C7—C6—C8—O3	168.4 (3)	Zn1^iii^—N4—C21—C22	−179.7 (3)
C10—N1—C9—N2	0.3 (3)	N4—C21—C22—N3	0.2 (5)
Zn1—N1—C9—N2	−174.91 (19)	C20—N3—C22—C21	0.2 (5)
C11—N2—C9—N1	0.1 (4)	C19—N3—C22—C21	172.2 (4)

Symmetry codes: (i) *x*−1, *y*, *z*; (ii) *x*, *y*, *z*+1; (iii) *x*, *y*, *z*−1; (iv) *x*+1, *y*, *z*.

### Hydrogen-bond geometry (Å, º)

**Table:**

*D*—H···*A*	*D*—H	H···*A*	*D*···*A*	*D*—H···*A*
C9—H9···O3^v^	0.93	2.38	3.188 (4)	145
C11—H11···O4^vi^	0.93	2.54	3.413 (4)	157
C14—H14···O1^vi^	0.93	2.38	3.306 (7)	171
C19—H19*B*···O2^vii^	0.97	2.38	3.200 (4)	142
C20—H20···O1^iii^	0.93	2.34	3.016 (4)	130
C21—H21···O4^viii^	0.93	2.54	3.092 (6)	119

Symmetry codes: (iii) *x*, *y*, *z*−1; (v) −*x*+1, −*y*+1, −*z*+2; (vi) −*x*, −*y*+2, −*z*+2; (vii) −*x*, −*y*+1, −*z*+2; (viii) *x*−1, *y*, *z*−1.
